# Overexpression of the Potato Monosaccharide Transporter *StSWEET7a* Promotes Root Colonization by Symbiotic and Pathogenic Fungi by Increasing Root Sink Strength

**DOI:** 10.3389/fpls.2022.837231

**Published:** 2022-03-24

**Authors:** Elisabeth Tamayo, David Figueira-Galán, Jasmin Manck-Götzenberger, Natalia Requena

**Affiliations:** Molecular Phytopathology, Botanical Institute, Karlsruhe Institute of Technology (KIT), Karlsruhe, Germany

**Keywords:** potato, SWEET transporters, arbuscular mycorrhizal symbiosis, *Rhizophagus irregularis*, *Fusarium oxysporum*

## Abstract

Root colonization by filamentous fungi modifies sugar partitioning in plants by increasing the sink strength. As a result, a transcriptional reprogramming of sugar transporters takes place. Here we have further advanced in the characterization of the potato SWEET sugar transporters and their regulation in response to the colonization by symbiotic and pathogenic fungi. We previously showed that root colonization by the AM fungus *Rhizophagus irregularis* induces a major transcriptional reprogramming of the 35 potato SWEETs, with 12 genes induced and 10 repressed. In contrast, here we show that during the early colonization phase, the necrotrophic fungus *Fusarium solani* only induces one SWEET transporter, *StSWEET7a*, while represses most of the others (25). StSWEET7a was also induced during root colonization by the hemi-biotrophic fungus *Fusarium oxysporum* f. sp. *tuberosi*. StSWEET7a which belongs to the clade II of SWEET transporters localized to the plasma membrane and transports glucose, fructose and mannose. Overexpression of *StSWEET7a* in potato roots increased the strength of this sink as evidenced by an increase in the expression of the cell wall-bound invertase. Concomitantly, plants expressing *StSWEET7a* were faster colonized by *R. irregularis* and by *F. oxysporum* f. sp. *tuberosi*. The increase in sink strength induced by ectopic expression of *StSWEET7a* in roots could be abolished by shoot excision which reverted also the increased colonization levels by the symbiotic fungus. Altogether, these results suggest that AM fungi and *Fusarium* spp. might induce *StSWEET7a* to increase the sink strength and thus this gene might represent a common susceptibility target for root colonizing fungi.

## Introduction

Sugar transport proteins play a crucial role in the long-distance distribution of carbohydrates throughout the plant. Photosynthates from source tissues have to be transported to net importer organs such as young leaves, reproductive structures, roots or tubers, in general known as sinks (Williams et al., 2000). And thus, a complex regulation of importers and exporters is required to guarantee the coordinated sugar partitioning that meets each organ demands. However, plant colonization by microbes creates new sinks, significantly altering sugar partitioning and sugar related metabolic activities (Doidy et al., 2012). Depending on the trophic mechanism exerted by microbes they might exert extensive tissue maceration leading to the release of the cell content including sugars (necrotrophs) or develop sophisticated mechanisms to divert nutrients toward the colonization interface (biotrophs). However, carbohydrates play a dual role in plant microbial interactions because they are necessary to cover the energetic costs of the defense responses and to sustain microbial growth. Therefore, carbohydrate reprogramming is intrinsic to plant-microbial compatibility.

There is accumulating evidence that sugar allocation might be tightly linked to the control of plant defenses and susceptibility (Moore et al., 2015; Hacquard et al., 2016; Yamada et al., 2016; Gebauer et al., 2017). For instance, Moore et al. (2015) showed that the partial resistance of wheat toward multiple pathogens in the locus Lr67 was due to mutations in a high affinity glucose transporter from the STP13 cluster. Furthermore, Yamada et al. (2016) showed that in *Arabidopsis* the regulation of the STP13 transporter activity is key for the resistance to bacterial pathogens. Another example showing how sugar availability modifies plant defense responses has been reported for the double mutant *SWEET11*/*SWEET12* in *Arabidopsis* (Gebauer et al., 2017). This mutant, impaired in sugar loading into the phloem, exhibits reduced susceptibility toward the infection by the fungus *Colletotrichum higginsianum.* Increased resistance was mediated by activation of the salicylic acid-mediated defense response (Gebauer et al., 2017). Although there are several other examples of sugar transporter activation by microbes that potentially implicate changes in susceptibility toward those microbes, perhaps the most prominent example is the activation of rice SWEET transporters by bacteria of the genus *Xanthomonas* by transcriptional activator like (TAL) effectors (reviewed in Eom et al., 2015; Kim et al., 2021). Because even before knowing which genes were involved, mutations in SWEET genes were shown to be susceptibility targets toward those bacteria (Yang et al., 2006; Antony et al., 2010; Liu et al., 2011).

AM fungi are obligate biotrophs, requiring the plant for their carbon nutrition, provided in form of monosaccharides and lipids (Shachar-Hill et al., 1995; Helber et al., 2011; Bravo et al., 2017; Keymer et al., 2017; Luginbuehl et al., 2017). Consequently, and similar to other microbes colonizing plants, AM fungi impose a major reorganization in the carbon partitioning, and an increase in the sink strength of the colonized tissue (Wright et al., 1998; Graham, 2000; Boldt et al., 2011; Bitterlich et al., 2014). Furthermore, AM colonization affects the subsequent or parallel colonization by other microorganisms, and the defense capacities of the plant. For instance, AM fungi have been associated with reduction of incidence of several root rot and wilting phenotypes caused by several fungal species including *Fusarium*, as well as by several oomycetes (summarized in Whipps, 2004).

However, the protection they offer is not universal, and the magnitude depends on the AM species employed and on the environmental conditions (Pozo and Azcon-Aguilar, 2007). Liu and coworkers (Liu et al., 2007) using an Affymetrix microarray approach demonstrated that mycorrhizal colonization significantly alters gene expression in a local and systemic manner and demonstrated the induction of a functional defense response in shoots toward a pathogenic *Xanthomonas* spp. Moreover, a study in rice showed that the transcriptomic responses imposed by arbuscular mycorrhizal and pathogenic fungi on plants are partially overlapping, suggesting that root infecting fungi might have similar plant targets required for root infection (Guimil et al., 2005). However, the mechanisms behind are still elusive, and more studies are required involving complex interactions of AM fungi and pathogens on the same plant to investigate if modification of sugar partitioning could be involved in the altered defense responses of mycorrhizal plants.

Two models have been proposed to explain how sugars can modulate plant defense responses toward microbial pathogens (Bezrutczyk et al., 2018). In the first model, and given that the goal of every colonizing microbe is to obtain fixed carbon from its host, plants starve the pathogen for sugar thereby increasing resistance. The second model, which is not incompatible with the first one, proposes that specific sugar ratios at infection sites elicit defense responses that keep microbes at bay (Bezrutczyk et al., 2018). There is supporting evidence for both models in the literature, and likely it depends on many other factors such as the type of pathogen, the infected organ and the pathogenicity tools (i.e., effector secretion) of the given microbe. In any case, it is thus expected that reorganization of plant sugar transporter expressions will accompany each plant-microbial interaction.

In this context, it is thus conceivable that changes in susceptibility to pathogens observed in plants colonized by mycorrhizal fungi might be at least in part due to the changes in the carbon partitioning imposed by the symbiosis. We decided to start investigating this hypothesis by further characterizing the function of several SWEET transporters that were found induced during symbiosis between potato plants and the AM fungus *Rhizophagus irregularis* (Manck-Gotzenberger and Requena, 2016) and in particular by analyzing their involvement in the colonization by pathogenic fungi. SWEET transporters are bidirectional sugar facilitators (Chen L. Q. et al., 2015; Eom et al., 2015; Breia et al., 2021). They can be located either at the plasma membrane, at the tonoplast or in the Golgi membrane. Although their primary role in plants is to serve functions such as pollen nutrition, seed filling, flower and fruit development or phloem loading (Chen et al., 2010, 2012; Sosso et al., 2015; Yang et al., 2018), they are a paradigm of how microbes can modify the plant carbohydrate program toward their own benefit as shown above. Thus, SWEET transcriptional activation in response to TAL effectors from pathogenic bacteria such as *Xanthomonas* spp. allows those bacteria to access sugars particularly in tissues where sugar movement is symplastic (Yang et al., 2006; Chen et al., 2010; Yu et al., 2011; Cohn et al., 2014; Hu et al., 2014). Furthermore, disease resistance can be achieved if transcriptional activation is prevented for instance by mutations in the binding site of TAL effectors in the promoter of SWEET genes (Chen et al., 2010; Yu et al., 2011; Streubel et al., 2013).

Here we have analyzed the spatial induction of four mycorrhiza-induced SWEET genes in potato, their transport activity and their subcellular localization. The expression of all potato SWEETs in response to root colonization of the necrotrophic fungus *Fusarium solani* was also analyzed, and the results showed that only one SWEET gene, *StSWEET7a*, is induced, while the majority are downregulated. Furthermore, we present data about the functional role of *StSWEET7a*, commonly induced by both symbiotic and pathogenic fungi, using overexpression composite plants. Our results point toward a role of StSWEET7a at increasing the sink strength of colonized roots thereby facilitating the colonization by both types of microbes.

## Materials and Methods

### Biological Material and Growth Conditions

*Solanum tuberosum* cv. Desiree was propagated as cuttings axenically in plastic containers with Murashige and Skoog medium containing vitamins and 30 g/L sucrose (Murashige and Skoog, 1962) solidified with 3 g/L Phytagel (P8169, Sigma-Aldrich, Germany) at 21°C and 16 /8 h day/night rhythm.

For mycorrhizal colonization experiments, 2-week-old cuttings or transgenic composite plants were transferred to 80 ml falcons with a sand:gravel (1:4) mixture. Plants were inoculated by mixing the substrate with 2-month-old *Daucus carota* root cultures grown monoaxenically in association with *R. irregularis* DAOM 197198 (Schenck and Smith, 1982; Kruger et al., 2012) on M-medium with sucrose at 27°C in darkness (Bécard and Fortin, 1988). One Petri dish of carrot roots was used to inoculate two 80 ml falcons. Plants were grown at 25°C and 16/8 h light/darkness photoperiod and fertilized twice a week with 5 ml Murashige and Skoog nutrient solution with low phosphate content (50 μM). Non-mycorrhizal controls were treated the same. After 6 weeks (6 weeks post inoculation), a fraction of the roots was separated and stored in 1X PBS for further analysis and quantification of mycorrhizal colonization, while the rest of the roots and shoots were harvested separately, immediately frozen in liquid nitrogen and stored at −80°C until used.

*Nicotiana benthamiana* was used for transient expression of GFP fusion proteins in localization analyses. Plants were grown in soil at 28°C in a growth chamber (CLF Plant Climatics, Germany), with a 16/8 h of light/darkness photoperiod and watered on demand.

*Fusarium solani* strain BBA72084 (Malonek et al., 2005), was cultivated on CM at 28°C for 6 days to obtain microconidia (Talbot et al., 1993). *S. tuberosum* plants (3 weeks old) were inoculated with a spore suspension containing 5 × 10^6^ microconidia/ml, according to Di Pietro et al. (2001). Roots were harvested 48 h post inoculation.

*Fusarium oxysporum* f. sp. *tuberosi* (CABI culture collection, strain 141127^1^), was propagated on CM at 28°C. For the infection assays, roots of *S. tuberosum* cv. Desiree cuttings were immersed in a sterile 0.2% gelatine solution containing 5 × 10^6^ microconidia/ml for 30 min under gentle agitation. Plants were then transferred to falcons containing the above-mentioned sand:gravel mixture and grown at 25°C as described above, but fertilized with Murashige and Skoog nutrient solution with full phosphate concentration (1.25 mM). Plants were harvested at 9 days or 7 weeks post inoculation and immersed in 1X PBS to visualize infection using WGA-FITC as described in Rech et al. (2013).

### Gene Isolation and Constructs

For growth assays in *Saccharomyces cerevisiae*, the full-length cDNAs of *StSWEET1b*, *2c*, *7a*, and*12a* were amplified from cDNA with *Pst*I and *Xho*I restriction sites and subcloned into pCR8/GW/TOPO (Invitrogen by Thermo Fisher Scientific, Germany), to afterward clone them into pDR196, which contains a fragment of the plasma membrane ATPase promoter (Rentsch et al., 1995). The full-length cDNA of *Htx2* yeast gene was also cloned into pDR196 and used as positive control in the complementation analyses of the EBY.VW4000 mutant strain (Wieczorke et al., 1999).

For localization analyses in *N. benthamiana*, the ORFs of *StSWEET1b*, *2b*, *2c*, *7a*, and *12a* without stop codon were amplified from cDNA and subcloned into pENTR/D-TOPO (Invitrogen by Thermo Fisher Scientific, Germany). Afterward, the constructs were cloned into the destination vector pCGFP-RR for a C-terminal GFP-tagging (Kuhn et al., 2010).

For overexpression analyses in *S. tuberosum* roots, the open reading frame of *StSWEET7a* was amplified from gDNA, subcloned into pENTR/D-TOPO (Invitrogen by Thermo Fisher Scientific, Germany) and cloned into the destination vector 2xP35S-pKGW-RedRoot (Heck et al., 2016).

All primers used for cloning of the constructs are listed in Supplementary Table 1.

### Promoter Analysis

For promoter-reporter assays, 2 kb fragments of the *StSWEET1b*, *2c*, *7a*, and *12a* promoters were cloned into the Gateway binary vector pPGFPGUS-RR as described in Manck-Gotzenberger and Requena (2016). *Agrobacterium rhizogenes*-mediated transformation of *S. tuberosum* and mycorrhizal inoculation with *R. irregularis* was carried out.

### Yeast Growth Assays

Yeasts were transformed with the corresponding constructs using a lithium acetate-based method (Gietz and Woods, 2002), and transformants were selected in SD medium by autotrophy to uracil. For the complementation assay, the yeast strain EBY.VW4000 (Wieczorke et al., 1999) and SD media supplemented with fructose, galactose, glucose, and mannose were used.

For the complementation assay, the medium contained 1.5% gold agar (Affymetrix). Yeast cultures were grown in SD liquid medium without uracil and supplemented with 2% maltose were diluted to an optical density at 600 nm of 0.4 and then grown to exponential phase (OD_600_ 0.8–1). Cultures were washed twice with distilled water and brought to an optical density at 600 nm of 1. Serial 1:10 dilutions were spotted (5 μl) onto plates containing the different sugars to determine transport as compared with strains transformed with the empty vector (EV).

### Transient Expression of Proteins in *Nicotiana benthamiana*

The plasmids constructed for localization analyses were transformed into *Agrobacterium tumefaciens* GV3101 strain. A bacterial suspension was infiltrated into leaves of 3-week-old *N. benthamiana* plants using a needleless syringe and the p19 protein of tomato bushy stunt virus was used to suppress gene silencing in co-transformation, after the protocol of Voinnet et al. (2003). Cells were kept overnight in the dark in infiltration media [2% sucrose solution 10 mM MgCl_2_, 10 mM MES-KOH, 150 μM acetosyringone in dimethyl sulfoxide (DMSO)], with an OD_600_ of 0.8–1 at room temperature before co-infiltration with a ratio of 1:1. After infiltration of the cultures into the underside of two-three leaves per plant, plants were incubated at 21°C in a low illuminated place and confocal microscopy was performed 2–3 days after infiltration.

### *Agrobacterium rhizogenes*-Mediated Transformation

*Agrobacterium rhizogenes* ARquaI containing the appropriate vector was used for the root transformation of *S. tuberosum* cv. Desiree. *S. tuberosum* composite plants were generated by stab inoculation after (Horn et al., 2014). For this purpose, 2-week-old cuttings were transferred to fresh potato medium slants without sucrose in 15 cm Petri dishes. The plants were stabbed into the second or third internode from the roots with an inoculation needle dipped into the agrobacteria. Two weeks after transformation, the wild-type roots were cut and the *S. tuberosum* cuttings were washed two times in water supplemented with 600 mg/L Augmentin (AmoxiClav, Hikma Farmaceutical, Portugal) and 200 mg/L Cefotaxime (Actavis GmbH & Co. KG, Langenfeld, Germany) and transferred to fresh potato medium slants without sucrose supplemented with 400 mg/L Augmentin and 200 mg/L Cefotaxime. Transformed roots were visualized using the DsRed marker under the fluorescence binocular. Transformed plants were transferred to 80 ml pots 4 weeks after transformation.

### Microscopy and Image Processing

Confocal microscopy images were taken using a Leica TCS SP5 (DM5000) confocal microscope with conventional PMT detectors and the color camera Leica DFC295, using the LASAF v2.6 software. The fluorescent proteins eGFP (488 nm) and WGA-FITC (488 nm) were excited with an argon laser while DsRed (561 nm) was excited with a DPSS laser. Emission of eGFP was detected from 493 to 530 nm and DsRed from 566 to 670 nm. Emission of WGA-FITC was collected from 505 to 525 nm after excitation at 494 nm (argon laser). Pictures were processed using ImageJ 1.51n.^2^

### Morphometric Analysis of Composite Plants

For morphometric analysis of leaf surface and shoot branching, images of plants were taken and analyzed using AutoCAD 2010 (Autodesk, Inc., San Rafael, CA, United States). For this purpose, pictures of five plants from each treatment were scaled taking into account the length of the plate (15 cm). Five leaves of each plant were used for further calculations.

### Quantification of Mycorrhizal Colonization

Fungal structures were immunostained with WGA-FITC as described in Rech et al. (2013) for phenotypical analysis and quantification of mycorrhizal colonization. Quantification of mycorrhizal structures was carried out according to Trouvelot et al. (1986). F% represents the frequency of mycorrhization, M% the intensity of colonization, A% the abundance of arbuscules, and I% the abundance of hyphae in the root system. Vesicle quantification was carried out counting the number of vesicles present along at least 68 fields of vision per biological replicate observed with the 10× magnification at the confocal microscope (Leica TCS SP5, DM5000) and expressed as percentage of vesicles per root segment. Roots of four biological replicates per treatment (EV mycorrhizal and *StSWEET7a* OE mycorrhizal) were analyzed after immunostaining of *R. irregularis* with WGA-FITC.

### Quantification of Pathogen Colonization

To quantify the *F. oxysporum* f. sp. *tuberosi* colonization at 7 weeks post inoculation (wpi) in EV and *StSWEET7a* overexpressing roots, the fungus was stained with WGA-FITC and confocal microscopy pictures from different root regions for each treatment (four biological replicates each) were taken, with *n* ≥ 17 (*n*, number of root sections analyzed for each treatment). Using Fiji, the root surface areas were outlined manually and the fungal area was determined by applying a Minimum threshold to the FITC fluorescence channel (Schindelin et al., 2012). The extent of colonization was calculated as the fraction of root surface area covered by fungus.

### Gene Expression Analyses

Total RNA was extracted using the innuPREP RNA Kit (Analytik Jena AG). cDNA was synthesized as described in Kuhn et al. (2010) with the reverse transcriptase SuperScript II (Invitrogen, United States). Control PCRs were carried out using the *StActin* gene (XM_006345899) to check for the absence of genomic DNA contamination in the cDNA samples. Real time expression analyses were carried out using an iCycler MyIQ (Bio-Rad, United States) and MESA Green 231qPCR Master Mix Plus (Eurogentec, Germany) with 3–5 independent biological replicates depending on the experiment. Expression of *StActin* gene was used for normalization of the expression of plant genes as well as of *RiTEF* (DQ282611). Fugal genes’ expression was normalized to *RiTEF* transcript levels. *StPT4* (AY793559), *StInvCD141* (Z22645), *RiTEF*, *RiMST2* (HM143864), and *StFatM* (PGSC0003DMP400059797) were used as indicators of symbiosis status. The PCR program consisted in a 1 min incubation at 95°C, followed by 40 cycles of 30 s at 95°C, 30 s at 56°C and 30 s at 72°C, where the fluorescence signal was measured. The specificity of the PCR amplification procedure was checked with a heat-dissociation protocol (from 57 to 95°C) after the final cycle of the PCR. Oligonucleotides used can be found in Supplementary Table 1.

### Determination of Phosphate Concentration

Potato shoot phosphate concentration was determined with the *Malachite Green Phosphate Assay Kit* from Sigma. Frozen tissue was first homogenized using a mixer mill (three rounds of 1 min each with a frequency of 25 oscillations per second). A known amount of frozen tissue powder was collected in a separate tube to extract the phosphate from and later relativize the phosphate amount to the corresponding collected weight. Phosphate isolation was achieved through the incubation of the samples with 250 mM NaOH (sodium hydroxide) for 1 min at 95°C and later with an added equivalent volume of 250 mM HCl (hydrochloric acid, for pH neutralization) for 2 min at 95°C. Samples were then centrifuged to precipitate cell debris and supernatants containing the isolated phosphate were collected into new tubes. The determination of the phosphate concentration was conducted according to manufacturer instructions. Absorbances (λ = 620 nm) were measured using a plate reader (Tecan Infinite M Nano, Männerdorf, Switzerland) in two replicates. A calibration curve was prepared according to the manufacturer specifications and samples were diluted with Milli-Q water to produce absorbances within the range of the calibration curve.

### Statistical Analyses

Data shown in Figure 5A represent the mean of five biological replicates and error bars correspond to the standard deviation. For the rest of the figures showing graphs, boxplots were used to represent the data. In those, the mean is shown by an “x” and the median by a horizontal line while each dot represents the individual value for each biological replicate. For each parameter analyzed, each treatment was first subjected to the Shapiro–Wilk test for normality. If treatments were normally distributed, a two-tailed Student’s *T*-test was applied. In case one of the treatments (or both) were not normally distributed, a Mann–Whitney U test was applied. Significance is indicated by asterisks (^∗^*p* < 0.05; ^∗∗^*p* < 0.01) or “ns” (non-significant, *p* ≥ 0.05). The number of biological replicates (*n*) is indicated in each of the corresponding figure legend. Additional statistical analyses were carried out for data in Figures 4, 6 and Supplementary Figure 1 that are shown in Supplementary Tables 2, 3. For data comparing more than two groups and showing no normal distribution, the Kruskal–Wallis test was carried out and the significance calculated according to the Mann–Whitney U test.^3^ If data were showing a normal distribution an ANOVA and a Tuckey *post hoc* test were carried out. Significant differences with *p* < 0.05 are shown with different letters.

**FIGURE 1:**
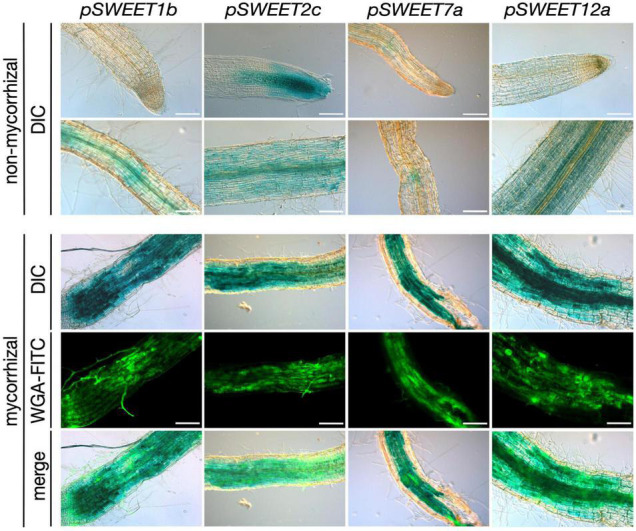
Promoter-reporter assay of *S. tuberosum SWEET* genes during AM symbiosis. A 2 kb fragment upstream of the ATG of the *S. tuberosum SWEET* genes *1b*, *2c*, *7a*, or *12a* was cloned in front of the GUS (β-*glucuronidase*) reporter gene and transformed in *S. tuberosum*. Composite plants were inoculated with *R. irregularis* and harvested 4 wpi (weeks post inoculation). β-Glucuronidase staining was carried out in non-mycorrhizal control roots and mycorrhizal roots. Fungal colonization was visualized by WGA-FITC (wheat germ agglutinin-fluorescein isothiocyanate) counterstaining labeling the fungal cell wall. Scale bars represent 200 μm. DIC, differential interference contrast; WGA-FITC signal is shown in green.

**FIGURE 2:**
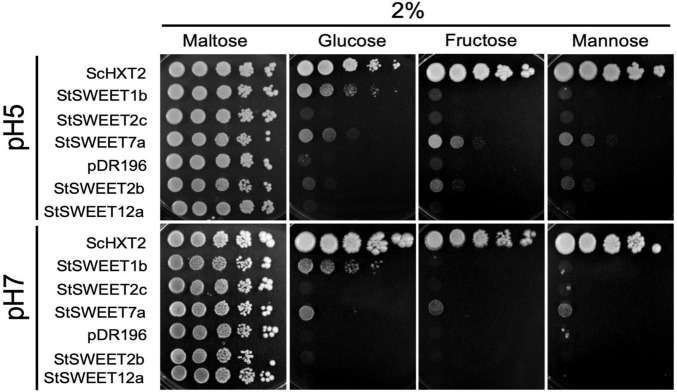
Analysis of the monosaccharide transport ability of five *S. tuberosum* SWEETs in yeast at pH 5 and pH 7. The EBY.VW4000 yeast strain was transformed with an empty vector (pDR196) or with the positive control *ScHXT2* or with the potato transporters *StSWEET1b*, *StSWEET2b*, *StSWEET2c*, *StSWEET7a*, or *StSWEET12a*, all cloned in pDR196. Strains were plated on SD medium without uracil supplemented with 2% maltose (growth control) or with 2% D-fructose, D-glucose, or D-mannose. Plates were incubated at 28°C for 5 days (pH 5) or for 12 days (pH 7).

**FIGURE 3:**
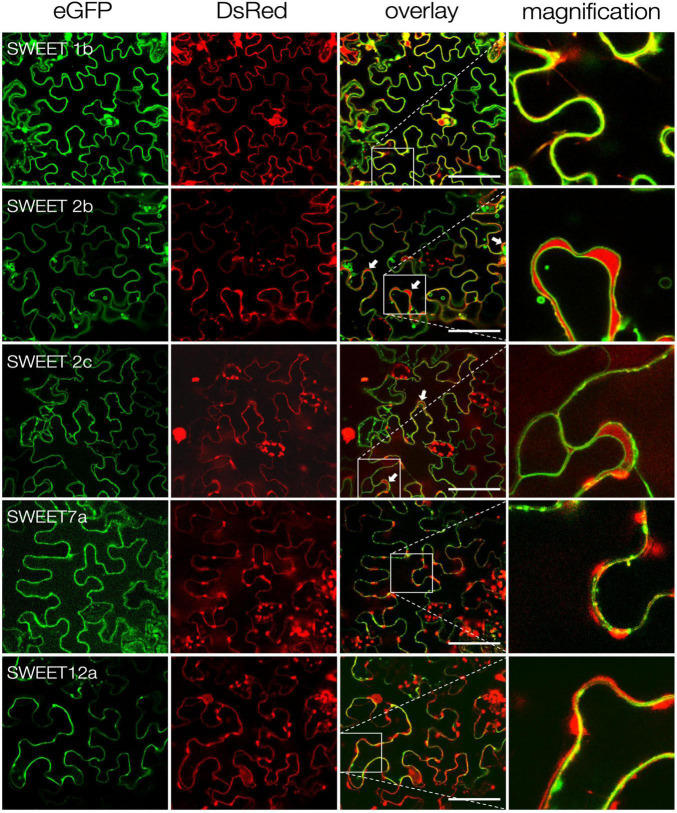
Subcellular localization of *S. tuberosum* SWEET proteins. Confocal imaging of ectopically expressed potato SWEET proteins fused to eGFP in *N. benthamiana* epidermal cells. Free DsRed was co-expressed as control for transformation and labels the cytoplasm and the plant nucleus. White arrows indicate cytoplasmic DsRed accumulations between the tonoplast and the plasma membrane. Zoom images of inlets are shown in the last column. Scale bars are 50 μm.

**FIGURE 4:**
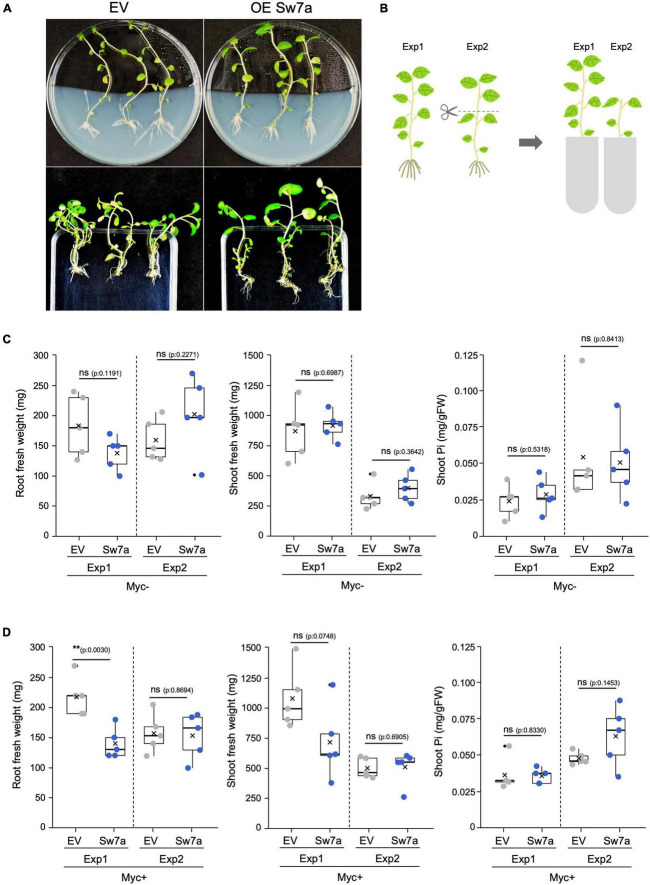
Effect in plants of the ectopic expression of *StSWEET7a*. **(A)** Ectopic expression of *StSWEET7a* in roots of composite potato plants modified shoot development as compared with plants transformed with an empty vector (EV). *StSWEET7a* expressing plants had larger leaves and less branches than EV plants before transplanting to pots. **(B)** Graphic representation of how Exp1 and Exp2 were carried out. Plants in both experiments were transplanted 4 weeks after transformation. In Exp1 plants were directly transplanted to pots, while in Exp2 plant shoots were excised right before transplanting. Plant growth parameters at the end of each experiment under non-mycorrhizal (Myc–) **(C)** and mycorrhizal (Myc+) conditions **(D)**. Five biological replicates (*n* = 5) were used for each treatment. Statistical significance (calculated as explained in section “Materials and Methods”) is shown with exact *p*-values and with asterisks, ns, non-significant, *p* > 0.05; *p < 0.05; **p < 0.01.

**FIGURE 5:**
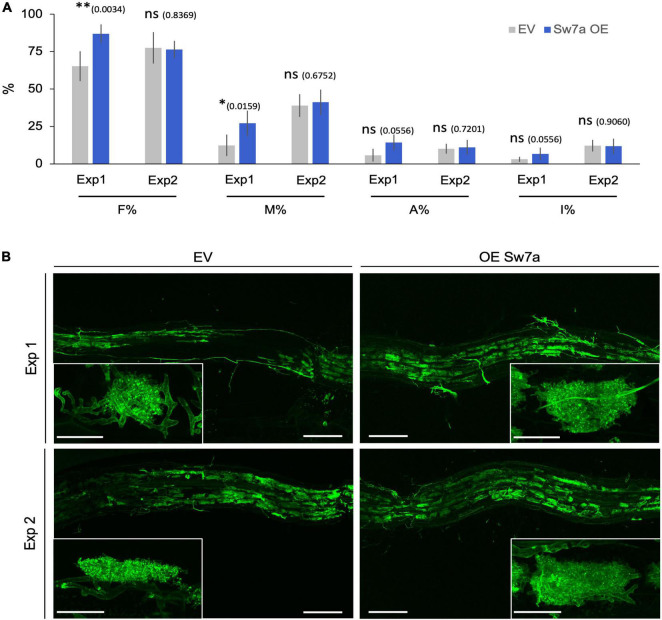
Ectopic expression of *StSWEET7a* modifies root colonization when shoot is intact. **(A)** Quantification of *R. irregularis* colonization was carried according to Trouvelot et al. (1986). F% represents the frequency of mycorrhization, M% the intensity of colonization, A% the abundance of arbuscules, and I% the abundance of hyphae in the root system. Five biological replicates (*n* = 5) were used for each treatment. Statistical significance (calculated as explained in section “Materials and Methods”) is shown with exact *p*-values and with asterisks, ns, non-significant, *p* > 0.05; ^∗^*p* < 0.05; ^∗∗^*p* < 0.01. **(B)** Representative pictures of the observed colonization in potato roots stained with WGA-FITC (green signal). Scale bars are 200 μm for overview pictures and 30 μm for pictures in inlets depicting single arbuscules.

**FIGURE 6:**
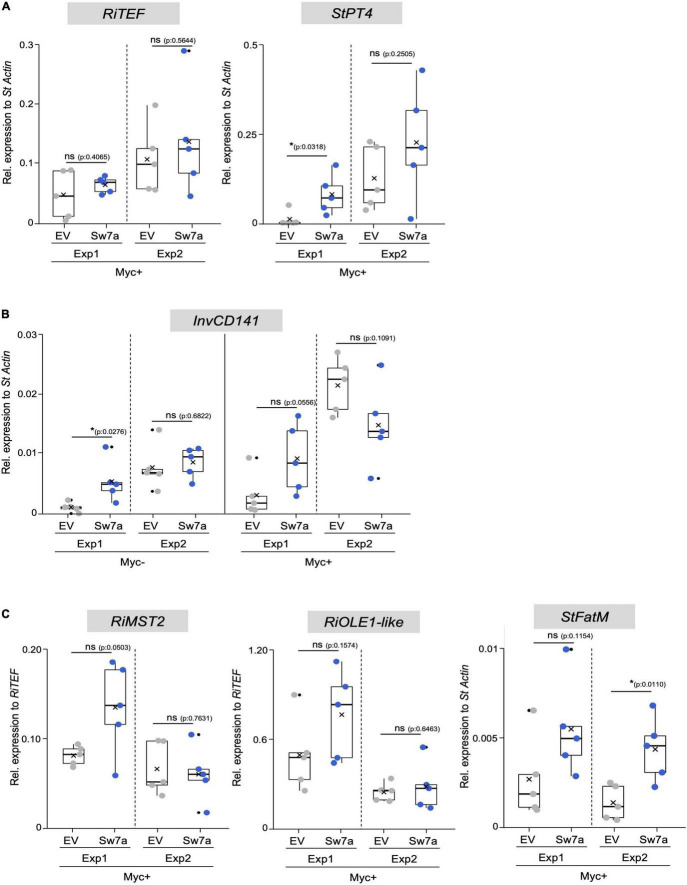
Gene expression analysis in potato roots ectopically expressing *StSWEET7a*. The impact of *StSWEET7a* overexpression on the colonization by *R. irregularis* was analyzed by qRT-PCR in roots of composite plants under the two experimental conditions (Exp1 and Exp2) using several symbiotic markers. **(A)** *RiTEF* and *StPT4* expression, **(B)** *StlnvCD141*, **(C)** *RiMST2*, *RiOLE1*-like, *StFatM*. Transcript levels were normalized to *StActin* in the case of plant genes and *RiTEF* and to *RiTEF* in the case of fungal genes. Statistical significance was calculated either using a two-tailed Student’s *T*-test or the Mann–Whitney U test, depending on the normality, as explained in section “Materials and Methods.” Significance is given by *p*. Exact *p*-values are shown, ns, non-significant, *p* > 0.05; *p < 0.05; **p < 0.01.

## Results and Discussion

### *SWEET* Promoter Activity Is Induced in Potato Roots in Arbuscule-Containing Regions

We had previously shown that root colonization by arbuscular mycorrhizal fungi induces a major transcriptional reprogramming affecting the expression of 22 of the 35 *SWEET* genes from potato, including the genes *StSWEET1b*, *2b, 2c*, *7a*, and *12a*. Furthermore, promoter-reporter analyses of three of these genes in the heterologous host *Medicago truncatula* showed promoter activity in arbuscule-enriched regions (Manck-Gotzenberger and Requena, 2016), suggesting they could be involved in carbohydrate regulation during symbiosis. Here, we analyzed the promoter activity of these genes in potato using the promoter-reporter GUS. Unfortunately, the promoter of *StSWEET2b* could not be amplified and was therefore not included in the assay. All these genes had basal expression levels in the cortex, sometimes also in root tips (*StSWEET2c*) under non-mycorrhizal conditions (Figure 1). However, transcript accumulation of *StSWEET1b*, *2c*, *7a*, and *12a* occurred in roots colonized by the AM fungus *R. irregularis* in areas enriched in arbuscules, and thus consistent with the localization data previously observed in *M. truncatula*. This result supports the hypothesis that these SWEETs could play a role in the mycorrhizal symbiosis by regulating the carbon partitioning in colonized roots and eventually increasing their sink capacity. Interestingly, putative orthologs of several of these genes in other plants have been shown to be also transcriptionally regulated during microbial interactions. Thus, for instance, the ortholog of potato *StSWEET1b* in *M. truncatula* was recently functionally characterized and also found to be strongly expressed in arbuscule-containing cells and to play a role in arbuscule maintenance (An et al., 2019). Furthermore, *MtSWEET1b* is also induced by nodulation (Kryvoruchko et al., 2016), supporting a symbiotic role for this transporter. *AtSWEET2* is induced in roots colonized by the pathogenic oomycete *Pythium* and its mutation reduces the resistance of plants to this pathogen (Chen H. Y. et al., 2015). The authors proposed this to be linked to a mechanism to prevent excess of glucose leakage out of roots. Three putative orthologs of potato *StSWEET7a*, the *M. truncatula MtSWEET6*, *Vitis vinifera VvSWEET7*, and the tomato ortholog *SlSWEET7b* have been also shown to be transcriptionally induced by microbes. *MtSWEET6* was induced in roots colonized either by AM fungi or by rhizobia (Kafle et al., 2019), further supporting our findings in potato (Manck-Gotzenberger and Requena, 2016). *VvSWEET7* transcription was increased in grapes in response to *Botrytis cinerea* infection (Breia et al., 2019), while *SlSWEET7b* was induced in roots of tomato upon infection with the root knot nematode *Meloidogyne incognita* (Zhao et al., 2018). *AtSWEET11* and *AtSWEET12* have been shown to be induced at infection sites during clubroot disease in order to deliver carbohydrate to the developing plasmodia and coincident with increases in apoplastic invertase and sucrose synthase gene expression (Walerowski et al., 2018). Collectively, these findings suggest that microbial colonization of plants imposes changes in carbon distribution that are facilitated by SWEET transporters.

### StSWEET1b and StSWEET7a Are Monosaccharide Transporters

Eom et al. (2015) had proposed that the phylogenetic classification of SWEETs in clades did not correlate with their physiological function (seed filling, pollen nutrition, etc.) but rather with their substrate preference. Thus, it was proposed that proteins from clade I and II (like StSWEET1b, 2b, 2c and 7a) would be monosaccharide transporters, while SWEETs in clade III (like StSWEET12a) would be more likely sucrose transporters. However, the specificity of SWEET transporters for mono- or disaccharides is sometimes controversial in the literature. Thus, while most SWEET transporters are specific for only one type of sugar, for some others it is not totally clear, and some have been shown to transport both, mono- and disaccharides (Kim et al., 2021). Furthermore, some SWEETs have been demonstrated to transport even sugar unrelated substrates, such as GA (Kanno et al., 2016; Morii et al., 2020).

In order to investigate the sugar transport activity of the mycorrhiza-induced potato SWEETs, complementation analyses in yeast were carried out. To that end, the hexose transport-deficient strain EBY.VW4000, that can only grow on monosaccharides if complemented with a functional plasma membrane-localized hexose transporter was used. The ability of the selected potato SWEET proteins to restore growth of this strain was then analyzed using different monosaccharides as single carbon sources. The full-length cDNAs of the *StSWEETs 1b*, *2b, 2c*, *7a*, and *12a* were expressed under the control of the constitutive promoter *PMA1* (coding for the yeast proton ATPase). As a positive control the yeast high affinity monosaccharide transporter ScHXT2, cloned in the same vector, was used. Growth of the transformed strains containing either one of the transporters or the EV was scored on minimal medium containing 2% of D-glucose, D-fructose, or D-mannose as single carbon sources. The results showed that the yeast strain expressing the potato *StSWEET1b* is able to grow on 2% D-glucose but it is not able to restore growth on any of the other monosaccharides analyzed (Figure 2). This is consistent with the results obtained for the *Arabidopsis thaliana* and *M. truncatula* orthologs, AtSWEET1 and MtSWEET1b, that have been shown to be specific glucose transporters (Chen et al., 2010; An et al., 2019).

In contrast to StSWEET1b, StSWEET2b and StSWEET2c, which also belong to clade I, were not able to complement the yeast growth on monosaccharides, although some residual growth was observed for StSWEET2b at pH 5, indicating that they might transport another substrate or that they are not localized at the plasma membrane (Figure 2). In fact, both SWEETs are in the same phylogenetic branch as AtSWEET2, a vacuolar transporter that likely transports glucose in and out of the vacuole to buffer the cytoplasmic content, and it is regulated in response to pathogens (Chen H. Y. et al., 2015). Similarly, *VvSWEET2a*, another transporter from this group from *V. vinifera*, is also induced by microbial colonization, suggesting that vacuolar control of sugar might be a critical issue for plants hosting microbes (Chong et al., 2014). Thus, we hypothesize that the regulation of StSWEET2b and 2c in potato by AM fungi might have an impact in the carbon partitioning of sugars among the cortical cells and allow arbuscule-containing cells to accumulate sugars in the vacuole to support the increased metabolic demands of these cells.

Similar to StSWEET1b, StSWEET7a was also able to transport glucose but in addition fructose and mannose (Figure 2). In all cases, the transport of these sugars was better at pH 5, while the glucose transport of StSWEET1b was equally good at both pHs. *V. vinifera* SWEET7, a putative ortholog of StSWEET7a, that is induced during infection with *B. cinerea*, was shown using radioactive sugar uptake measurements to also transport glucose and fructose, as well as polyols (Breia et al., 2019). Surprisingly, it also showed sucrose transport activity. However, the yeast strain used for these assays was also the EBYVW.4000 which contains the *SUC2* gene, coding for secreted and intracellular invertase (Carlson and Botstein, 1982). Therefore, the possibility that sucrose might be cleaved and taken up exists, and thus another strain with a deletion in *SUC2* could help to rule out that possibility (Helber et al., 2011). Potato StSWEET12a was unable to transport monosaccharides in the yeast assay and thus it might indicate that it is a sucrose transporter, similar to other SWEETs from clade III. We tested several other yeast strains to analyze the putative sucrose transport ability of StSWEET12a but results were not conclusive. Hence, other methods, such as the esculin uptake assay in yeast that was used to show the sucrose transport ability of StSWEET11 (Abelenda et al., 2019), are necessary.

As mentioned above, some SWEET transporters have shown to transport more than one substrate (including mono- and disaccharides) and for a few of them somewhat contradictory results have been shown. One example is the AtSWEET9, a sucrose transporter required for nectar production that was shown to be unable to transport glucose using the same yeast complementation assay employed here. However, this transporter exhibited a weak glucose transport activity when analyzed using FRET sensors (Lin et al., 2014). Also, the vacuolar transporter AtSWEET16 was shown to be able to take up mono- and disaccharides when expressed in *Xenopus* oocytes (Klemens et al., 2013). However, this is slightly contradictory to the experiments carried out by Guo et al. (2014). Using isolated *A. thaliana* vacuoles they could show that AtSWEET16 and the closely related AtSWEET17 exhibited similar phenotypes and sugar accumulation patterns in plants, but transport activity could be shown only for AtSWEET17 (Guo et al., 2014).

These results show the difficulty of these type of studies to ascertain the substrate and direction of transport for SWEET proteins and the need to establish further complementing methodologies.

### Subcellular Localization of Potato SWEETs

*In silico* analyses using WoLF Psort^4^ and the results above indicated that SWEETs 1b, 7a and 12a are likely plasma membrane transporters, while SWEET2b and 2c might be tonoplast transporters. In order to obtain more information about their subcellular localization, the corresponding proteins were tagged with GFP at their carboxy-terminus and expressed in *N. benthamiana* leaves. As positive control free eGFP, which localizes in the cytoplasm and in the nucleus, was also analyzed. In all cases, the DsRed fluorescent protein was used as transformation control, also showing localization in the cytoplasm and in the nucleus. Confocal microscopy analyses confirmed that, as predicted, StSWEETs 1b, 7a and 12a have plasma membrane localization (Figure 3). In contrast, StSWEETs 2b and 2c exhibited tonoplast localization, with visible accumulation of DsRed in the cytoplasm between tonoplast and plasma membrane (indicated with white arrows). These results could explain the inability of StSWEETs 2b and 2c to complement the mutant yeast strain, because they likely also localize at the tonoplast in the heterologous host.

### Overexpression of *StSWEET7a* in Roots Alters Sink Strength, Plant Architecture, and Mycorrhizal Colonization

In order to investigate the role of StSWEET7a during symbiosis, we deregulated its expression in roots by ectopically expressing the gene under the control of a constitutive promoter (*CMV 35S*) and carried out mycorrhizal colonization assays. Shoots of potato plant ectopically expressing *StSWEET7a* in roots had larger leaves, were less etiolated and survived better than control plants the adaptation to the substrate after transformation (Supplementary Figure 1). Furthermore, overexpressing plants developed less shoot branches than plants transformed with an EV (Figures 4A,B and Supplementary Figure 1). These differences in shoot development were, however, no longer visible at the end of the experiment, Exp1 (Figure 4C). However, under mycorrhizal conditions, *StSWEET7a* overexpressing plants developed significantly smaller roots (Figures 4C,D). In order to test whether these differences in growth at the end of the experiment were due to the larger size of the potato leaves and the reduced branching caused by the ectopic expression of *StSWEET7a*, a parallel experiment (Exp2) was carried out in which shoots from transformed plants were cropped before transplanting to the pots (Figure 4B). As expected, shoots from plants in Exp2 were smaller than those of Exp1, while root sizes were similar. Interestingly, the differences in root size observed in Exp1 between overexpressing plants and control plants (EV) were no longer visible when plants were colonized by *R. irregularis* (Figure 4D). This is similar of the conserved response of plants to decapitation that react with an increase in shoot branching mediated by sugars (Mason et al., 2014; Salam et al., 2021). This phenomenon has been recently explained as a competition for sucrose between the apical meristem and the axillary buds. The apical meristem acts as a strong sink depriving of sucrose the outgrowing buds and thus preventing branching (Barbier et al., 2015a,b). Taken together these results suggest that ectopic expression in roots of *StSWEET7a* alters the sugar partitioning in potato producing an increase in the root sink capacity as indicated by the change in shoot architecture, with a decrease in shoot branching and an increase in leaf size. In support of that, decapitation of all plants prior transplanting in Exp2 abolished the differences in plant architecture observed between control and *StSWEET7a* expressing plants in Exp1.

Plants ectopically expressing *StSWEET7a* in roots in Exp1 were faster colonized by *R. irregularis* than control plants (Figures 5A,B). Together with the decrease in root growth of those plants, this is reminiscent of the phenotype observed in the experiments of Bitterlich and coworkers, in which silencing of the *SlSUT2*, coding for a sucrose importer, increased mycorrhizal colonization but led to a shift in biomass from the plant to the fungus, thus abolishing the positive growth response of the mycorrhizal colonization (Bitterlich et al., 2014). In support of that hypothesis, the number of vesicles, fungal carbon storage structures, in roots expressing *StSWEET7a* was significantly higher than in EV plants (Supplementary Figure 1).

The higher mycorrhizal colonization of *StSWEET7a* plants in Exp1 paralleled to a higher expression of the symbiotic plant phosphate transporter *StPT4* but surprisingly not of the fungal translation elongation factor *RiTEF* (Figure 6A). This is interesting because an overexpression of *MtSWEET1b*, also a glucose transporter, led also to an increase in the mycorrhization levels in *M. truncatula* when inoculated by *R. irregularis* (An et al., 2019). However, this increase was also reflected in a higher *RiTEF* expression but not on a higher *MtPT4* expression (An et al., 2019), suggesting that MtSWEET1b and StSWEET7a despite transporting the same substrate are not fully redundant to each other. An and coworkers hypothesized that a higher MtSWEET1b activity could fuel monosaccharides toward the apoplast increasing fungal growth (An et al., 2019). Our results for StSWEET7a rather suggest that its role could be to support arbuscule functioning. However, we could not observe any significant change in the amount of phosphate accumulated in shoots between *StSWEET7a* overexpressing and control plants (Figure 4D). Another interesting observation of An et al. (2019) was that while impairment of the transport activity of MtSWEET1b did not alter mycorrhizal colonization, the expression of a dominant negative form accelerated arbuscule turn over and decreased *MtPT4* expression. This suggests that MtSWEET1b is able to oligomerize with itself or with other SWEETs, as it has been demonstrated for AtSWEET1 (Xuan et al., 2013). Therefore, it is tempting to speculate that StSWEET1b and StSWEET7a, which are both induced in arbuscule-containing cells in potato, could be acting as a dimer controlling glucose transport during symbiosis.

Interestingly, shoot decapitation prior transplanting in Exp2 accelerated mycorrhizal colonization as compared to Exp1, but eliminated the advantage given by the ectopic expression of *StSWEET7a*, and all plants were equally well colonized (Figures 5A,B). In agreement, *StPT4* levels were in general higher in Exp2 but there were no significant differences between treatments (Figure 6A).

In order to investigate whether the increased colonization in roots observed in Exp1 in response to the expression of *StSWEET7a* was related to changes in the carbon partitioning, the expression of the cell wall-bound invertase, that cleaves sucrose into glucose and fructose in the apoplast of sink organs, was analyzed in roots. Cell wall-bound invertases are essential enzymes for apoplastic phloem unloading and sink induction (Proels and Huckelhoven, 2014) and their expression and activity are known to be induced during symbiosis with arbuscular mycorrhizal fungi (Schaarschmidt et al., 2006). This induction takes place in and around arbuscule-containing cells, indicating that colonized cells act as a new sink organ. In potato, induction of *StInvCD141* gene expression paralleled to the expression of the mycorrhiza-induced SWEETs, including *StSWEET7a* (Manck-Gotzenberger and Requena, 2016). Here we could show that ectopic expression of *StSWEET7a* in roots significantly induces *StInvCD141* transcript accumulation under non-mycorrhizal conditions (Figure 6B), consistent with the hypothesized higher sink strength in those roots. In mycorrhizal plants, which already have a higher level of invertase expression, *StSWEET7a* also promoted transcript accumulation of *StInvCD141*, albeit not significantly. As we have shown above, StSWEE7a is able to transport glucose and fructose, and thus, we speculated that its constitutive expression in roots depletes the apoplast of those sugars inducing the cell wall-bound invertase what in turn boosts the unloading of sucrose from the phloem. A similar situation has been observed in tomato sink leaves, where SlSWEET1a fuels the unloading of sucrose from the phloem by taking up hexoses into phloem parenchyma (Ho et al., 2019). In agreement with our hypothesis that shoot decapitation prior transplanting increased sink strength in roots, invertase expression was higher in Exp2 than in Exp1 in all treatments, but differences between control and *StSWEET7a* overexpressing plants were no longer visible (Figure 6B).

We next investigated how these changes affected the expression of other symbiotic markers and, in particular, how the transport of fixed carbon toward the fungus could be affected. AM fungi are known to take up sugars, in the form of monosaccharides, and lipids from cortical cells (Choi et al., 2018). Thus, *RiMST2*, a fungal monosaccharide transporter only expressed during symbiosis was shown to import glucose and be critical for arbuscule integrity (Helber et al., 2011). Furthermore, AM fungi are fatty acid auxotrophs (Wewer et al., 2014), and arbuscules have been also shown to be nourished by monoacyl fatty acids synthesized in arbuscule-containing cells with the concomitant participation of several mycorrhiza-specific enzymes (Pimprikar et al., 2016; Bravo et al., 2017; Luginbuehl et al., 2017). We therefore analyzed the expression of two fungal markers, *RiMST2*, and *RiOLE1-like*, an acyl-CoA desaturase responsible for the synthesis of the unusual fatty acid, palmitvaccenic acid (16:1^Δ11cis^) which is characteristic of AM fungi (Olsson et al., 1995; Graham, 2000; Brands et al., 2020; Cheeld et al., 2020). In addition, and the expression of the plant marker *StFatM*, the ortholog of *MtFatM*, the plastidial acyl-ACP thioesterase mycorrhiza specific and required for lipid accumulation in the fungus was also investigated (Bravo et al., 2017; Brands et al., 2018). All three markers were transcriptionally more elevated in plants expressing *StSWEET7a* in Exp1, although none of them significantly, suggesting a higher metabolic activity in those roots (Figure 6C). Surprisingly, while both fungal genes were no longer regulated in Exp2, expression of *StFatM* was significantly induced in plants expressing *StSWEET7a*, although colonization in those plants was identical to control plants (Figure 5B). Taken together, these results suggest that the increase of the sink capacity mediated by the overexpression of *StSWEET7a* leads to an increased transport of fixed carbon toward the fungus boosting arbuscule development in the root.

### The Pathogen *Fusarium solani* Represses Most Potato *SWEET* Genes in Roots but Induces *StSWEET7a*

That overexpression of *StSWEET7a* in mycorrhizal potato roots led to an increase in the colonization of roots prompted us to investigate how the colonization by a pathogenic fungus would be affected in those plants. Sugars in the apoplast might play a dual role (Bezrutczyk et al., 2018). On one hand they serve as food for pathogens, and thus plants will try to downregulate sugar export while pathogens will seek the opposite. And on the other hand, apoplastic sugars have been shown to act as signals inducing defense reactions (Herbers et al., 1996; Herbers and Sonnewald, 1998). And hence both, the plant and the pathogen, might try to regulate sugar export/import to induce or prevent the elicitation of defense reactions. It is therefore not surprising that in addition to SWEET transporters, pathogens also induce monosaccharide transporters that likely try to compensate leakage of sugars to the microbial side (Fotopoulos et al., 2003; Lemonnier et al., 2014; Moore et al., 2015).

**FIGURE 7:**
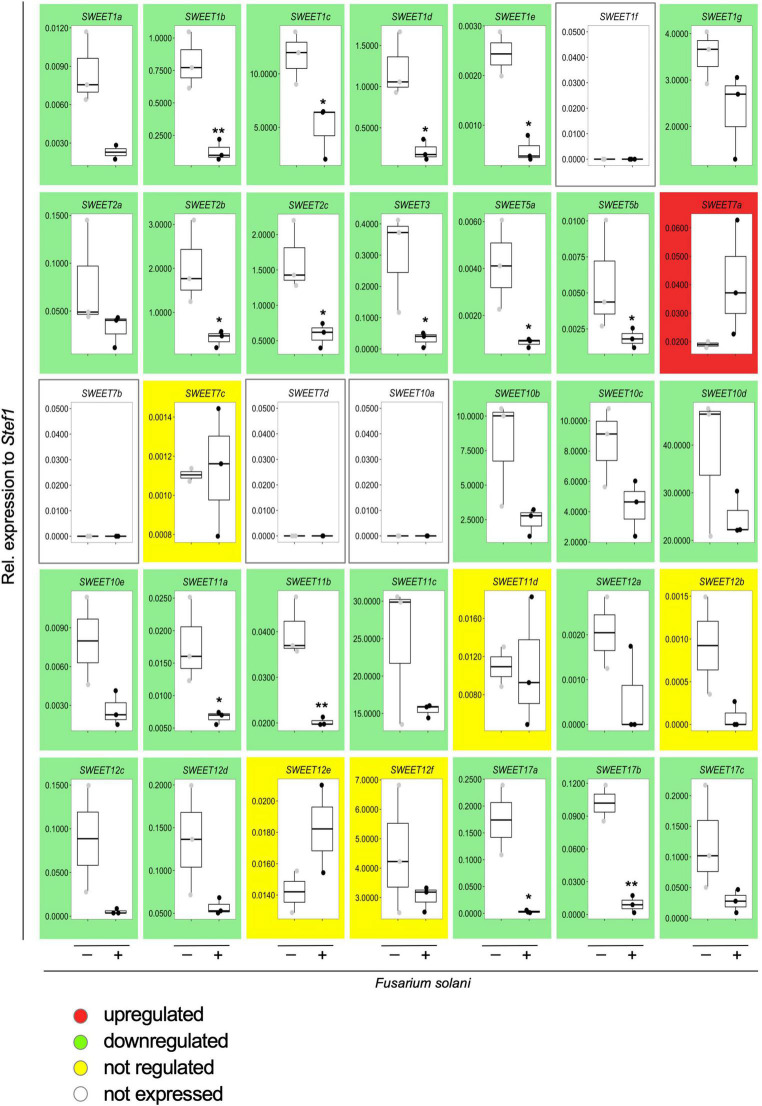
Effect of the infection by the necrotrophic fungus *Fusarium solani* on the expression of potato *SWEET* genes. Transcript levels of all potato *SWEET* genes in response to infection by *F. solani* were analyzed by qRT-PCR and normalized to *StTEF1*. Background color indicates whether the gene at issue is upregulated (red), downregulated (green), not regulated (yellow), or not expressed (white) in response to infection with *F. solani*. Pairwise comparisons were done with a two-tailed Student’s *T*-test. Significance is indicated with asterisks, *p < 0.05 or **p < 0.01.

Therefore, we first analyzed the expression of all potato *SWEETs* in response to *F. solani*. This necrotrophic fungus is the cause of potato tuber rot and can rapidly colonize the root system of *S. tuberosum*. We inoculated potato roots with *F. solani* spores and collected samples after 48 h when the fungus was extensively developing in the apoplastic spaces of the root cortex, but necrosis has not yet set on, and analyzed *SWEET* expression (Figure 7). Surprisingly, and in contrast to the reprogramming of SWEET genes observed during mycorrhizal colonization that included many upregulated genes (Manck-Gotzenberger and Requena, 2016), inoculation with *F. solani* repressed the expression of the majority of *SWEET* genes, including *StSWEET1b, 2b, 2c and 12a* (Figure 7). This is reminiscent of the situation observed when tomato cotyledons were infected with the necrotrophic fungus *B. cinerea* (Asai et al., 2016). In that experiment, most of the *SWEET* transporters were donwregulated in the pre-necrotic stage and only one transporter *SlSWEET15* was induced in response to *B. cinerea*. The authors suggested that *B. cinerea* uses SlSWEET15 to obtain glucose and sucrose from living tomato cells prior inducing the necrotrophic stage (Asai et al., 2016). In contrast, the reason why so many other transporters were downregulated, as it is also the case in our experiment is not clear. It is possible that either plants try to limit the amount of sugar released to the apoplastic space to limit pathogen development, or to change the hexose/sucrose ratio to elicit defense reactions as mentioned above. But alternatively, this repression might be mediated by the pathogen in order to prevent such defense reactions.

**FIGURE 8:**
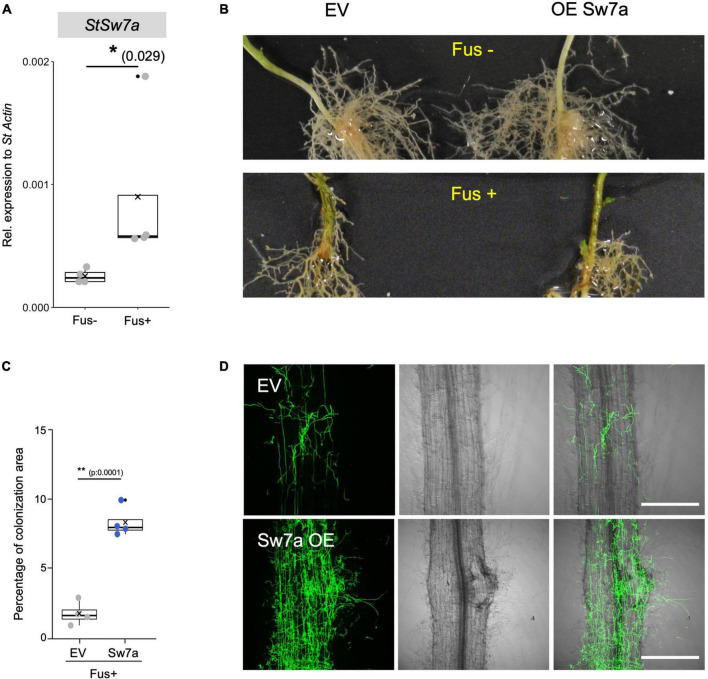
Ectopic expression of *StSWEET7a* accelerates potato root colonization by *Fusarium oxysporum* f. sp. *tuberosi*. **(A)** Colonization of potato roots by *F. oxysporum* f. sp. *tuberosi* significantly induces expression of *StSWEET7a* as shown by qRT-PCR normalized to *StActin*. Four biological replicates were analyzed (*n* = 4). Significance was calculated with a Mann–Whitney U test and represented by an asterisk, *p < 0.05. **(B)** Symptoms caused by *F. oxysporum* f. sp. *tuberosi* (Fus+) at the base of the shoot in both EV and *StSWEET7a* composite plants are shown as compared to non-infected plants (Fus–). **(C)** The extent of colonization by *Fusarium* was calculated as the fraction of root surface area covered by fungus using the Fiji software after staining the fungus with WGA-FITC. Confocal microscopy pictures from different root regions for each treatment (four biological replicates each) were taken, with *n* > 17, (*n*, number of root sections analyzed for each treatment). **(D)** Representative confocal microscopy pictures showing the colonization of EV and *StSWEET7a* overexpressing roots by *F. oxysporum* f. sp. *tuberosi*. The fungus was stained using WGA-FITC (left, green channel; center, bright field; right, overlay). Scale bar corresponds to 200 μm.

Interestingly, the only potato *SWEET* gene that showed an upregulation in response to *F. solani* (albeit not significant) was *StSWEET7a* (Figure 7), suggesting that colonization by the pathogen might activate the expression of this transporter to provide the fungus with monosaccharides and/or to increase the sink strength of the root, as in the case of *B. cinerea* in tomato (*SlSWEET15*) or in *V. vinifera* (*VvSWEET7*) (Asai et al., 2016; Breia et al., 2019). We then went on to analyze the expression of *StSWEET7a* in potato plants colonized by *F. oxysporum* f. sp. *tuberosi*, a less aggressive pathogen than *F. solani* (Manici and Cerato, 1994), that rather behaves like a hemi-biotrophic fungus and takes longer to colonize the root and produces a variety of symptoms such as wilting, stem end rot, chlorosis, necrosis or damping-off (Majeed et al., 2018). Results showed that this species also induces the expression of *StSWEET7a* in roots (Figure 8A), suggesting that perhaps a common mechanism of induction might take place in all cases.

### Overexpression of *StSWEET7a* Accelerates Root Colonization by *Fusarium oxysporum* f. sp. *tuberosi*

To investigate the role of StSWEET7a in the *F. oxysporum* f. sp. *tuberosi* infection we inoculated plants ectopically expressing *StSWEET7a* in roots or control plants. Although all plants were colonized, there were no wilting symptoms at the end of the experiment. Only minor stem end rot symptoms occurred, but there were no differences between the two treatments (Figure 8B). This lack of wilting symptoms by some *F. oxysporum* f. sp. *tuberosi* strains causing dry rot is not unusual, and it has been previously reported (Tivoli et al., 1988; Manici and Cerato, 1994). However, the microscopic observation of roots, clearly showed their colonization by the fungus. Furthermore, it could be observed that increasing the expression of *StSWEET7a* in roots led to a faster colonization (Figures 8C,D and Supplementary Figure 2). Overall, these results support the hypothesis that induction of *StSWEET7a* by *Fusarium* serves the feeding of the fungus by increasing unloading of sucrose from the apoplast and thus mirroring the effect of root colonization by the symbiotic fungus *R. irregularis* as described above. In addition, we cannot exclude that StSWEET7a might directly feed root colonizing fungi by exporting monosaccharides at the places of colonization.

Similar results in other plants have been observed in response to ectopic expression of other *SWEET* genes. Thus, in rice, colonization by the necrotrophic *Rhizoctonia solani* induces the expression of *OsSWEET11*, and plants overexpressing this transporter were faster colonized than control plants (Gao et al., 2018). Also, *A. thaliana* plants in which *AtSWEET4* was deleted were more resistant to *B. cinerea* (Chong et al., 2014). However, this is not always the case, and in some cases, overexpression of several *SWEET* genes led to increased resistance toward certain pathogens. Thus, overexpression of *VvSWEET4* in *V. vinifera* led to a decreased susceptibility toward *Pythium irregulare* (Meteier et al., 2019). The authors explain this effect, which is contrary to the observations made by Chong et al. (2014) in the same group, by explaining that the rise in sugars in roots expressing *VvSWEET4* was responsible for the observed increase in flavonoid compounds that ultimately acted as antifungal agents (Meteier et al., 2019). Also, in sweet potato, resistance to *F. oxysporum* was induced in transgenic plants overexpressing *IbSWEET10* (Li et al., 2017). Here the authors suggested that reduction in sugar content (sucrose, glucose, and fructose) observed in leaves of the transgenic plants contributed to restrict the growth of the pathogen (Li et al., 2017).

## Conclusion

Sugar transport is the hub where plants and their colonizing microbes converge to regulate it in their own benefit and SWEET transporters are emerging as important players in this market. However, because plants are in nature not colonized by just one type of microorganism, it seems currently difficult to predict how induction or deletion of a specific SWEET gene might impact on the colonization of this plant by this or by other pathogenic/symbiotic microbes. This makes it difficult to use these transporters as targets to improve plant growth or resistance. Therefore, attention should be paid to different aspects such as the root organ in which SWEET transporters are induced by microbes, whether deregulation takes place systemically or in one specific organ, as well as the type of microbial interaction (biotrophic or necrotrophic). All these are key factors that need to be taken into account in a more systematic manner. An outstanding question is also how microbes regulate expression of *SWEET* genes by mechanisms other than those involving TAL effectors. Are there other effectors? Why are some transporters like StSWEET7a induced by pathogenic and symbiotic fungi? Do they use the same activation mechanisms? And in case plants are colonized by both microbes simultaneously, how will plant susceptibility be affected by the deregulation of StSWEET7a? Can we learn of these mechanisms to produce improved plants? These and many other questions remain yet unsolved and will require further research efforts.

## Data Availability Statement

The datasets presented in this study can be found either in the NCBI database or in the Solgenomics database.

## Author Contributions

NR, ET, and JM-G conceptualized the work. ET, JM-G, and DF-G carried out the experiments. NR wrote the manuscript with contributions from ET and DF-G. All authors contributed to the article and approved the submitted version.

## Conflict of Interest

The authors declare that the research was conducted in the absence of any commercial or financial relationships that could be construed as a potential conflict of interest.

## Publisher’s Note

All claims expressed in this article are solely those of the authors and do not necessarily represent those of their affiliated organizations, or those of the publisher, the editors and the reviewers. Any product that may be evaluated in this article, or claim that may be made by its manufacturer, is not guaranteed or endorsed by the publisher.

## Footnotes

1
https://www.cabi.org/products-and-services/bioscience-services/microorganism-supply-services/

2
http://fiji.sc/Fiji

3
https://www.Statskingdom.com

4
https://wolfpsort.hgc.jp

## Supplementary Material

Supplementary Figure 1 Ectopic expression of *StSWEET7a* in roots modifies plant architecture and survival after transplanting. **(A)** Relative expression of *StSWEET7a* (*StSw7a*) normalized to *StActin* was measured by qRT-PCR in roots of composite plants grown under two mycorrhizal conditions (Myc−, Myc+) and under two different experimental conditions (Exp1 and Exp2). Statistical significance was calculated using the Mann–Whitney U test. Significance is given by *p*-values. Exact *p*-values are given, ns, non-significant, *p* > 0.05; ^∗^*p* < 0.05; ^∗∗^*p* < 0.01. **(B)** Leaf surface and shoot branching were analyzed in plants ectopically expressing *StSWEET7a* as compared to EV plants. The number of biological replicates for each treatment for leaf surface analyses was five (*n* = 5) with five leaves analyzed per biological replicate. Six biological replicates per treatment were analyzed for root branching (*n* = 6). Statistical significance was calculated either using a two-tailed Student’s *T*-test (leaf surface) or the Mann–Whitney U test (shoot branching), depending on the normality, as explained in section “Materials and Methods.” Significance is given by *p*-values. Exact *p*-values are given, ns, non-significant, *p* > 0.05; ^∗^*p* < 0.05; ^∗∗^*p* < 0.01. **(C)** Survival percentage of transformed plants after transplanting to pots comparing plants expressing StSWEET7a in roots vs. EV plants. Significance was calculated according to the Kruskal–Wallis and each pair of groups was compared using the Mann–Whitney U test. Different letters indicate significance with *p*-value < 0.05. **(D)** Percentage of vesicles (as number of vesicles per root segment) of transformed plants expressing *StSWEET7a* in roots compared to EV plants. Statistical significance was calculated using a two-tailed Student’s *T*-test. ^∗^*p* < 0.05.

Supplementary Figure 2 Representative pictures of plant roots infected with *Fusarium oxysporum* f. sp. *tuberosi* in plants expressing *StSWEET7a* or an empty vector (EV). Confocal microscopy pictures showing *F. oxysporum* f. sp. *tuberosi* colonization 7 weeks post inoculation in four different transgenic lines for each treatment. The fungus was stained with WGA-FITC (left, bright field; center, green channel; right, overlay). Scale bar corresponds to 200 μm.
