# Supplementary figures and images for: Overexpression of the Potato Monosaccharide Transporter StSWEET7a Promotes Root Colonization by Symbiotic and Pathogenic Fungi by Increasing Root Sink Strength
Source: Front Plant Sci. 2022 Mar 24;13:837231. doi: 10.3389/fpls.2022.837231 (PMC8987980; doi:10.3389/fpls.2022.837231)

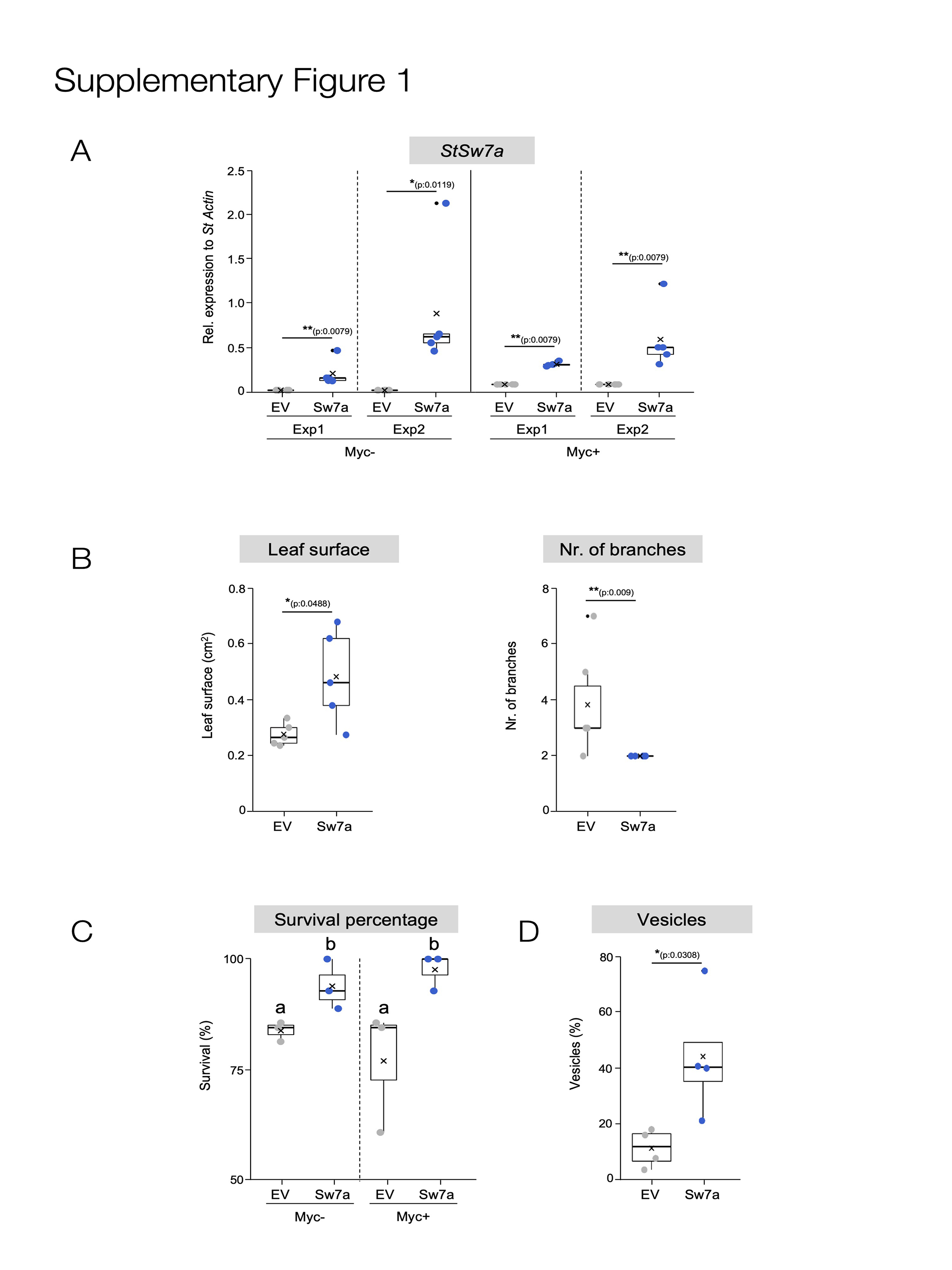

Supplement: Supplementary Figure 1 — Ectopic expression of StSWEET7a in roots modifies plant architecture and survival after transplanting. (A) Relative expression of StSWEET7a (StSw7a) normalized to StActin was measured by qRT-PCR in roots of composite plants grown under two mycorrhizal conditions (Myc−, Myc+) and under two different experimental conditions (Exp1 and Exp2). Statistical significance was calculated using the Mann–Whitney U test. Significance is given by p-values. Exact p-values are given, ns, non-significant, p > 0.05; ∗p < 0.05; ∗∗p < 0.01. (B) Leaf surface and shoot branching were analyzed in plants ectopically expressing StSWEET7a as compared to EV plants. The number of biological replicates for each treatment for leaf surface analyses was five (n = 5) with five leaves analyzed per biological replicate. Six biological replicates per treatment were analyzed for root branching (n = 6). Statistical significance was calculated either using a two-tailed Student’s T-test (leaf surface) or the Mann–Whitney U test (shoot branching), depending on the normality, as explained in section “Materials and Methods.” Significance is given by p-values. Exact p-values are given, ns, non-significant, p > 0.05; ∗p < 0.05; ∗∗p < 0.01. (C) Survival percentage of transformed plants after transplanting to pots comparing plants expressing StSWEET7a in roots vs. EV plants. Significance was calculated according to the Kruskal–Wallis and each pair of groups was compared using the Mann–Whitney U test. Different letters indicate significance with p-value < 0.05. (D) Percentage of vesicles (as number of vesicles per root segment) of transformed plants expressing StSWEET7a in roots compared to EV plants. Statistical significance was calculated using a two-tailed Student’s T-test. ∗p < 0.05. [file Image_1.jpeg]

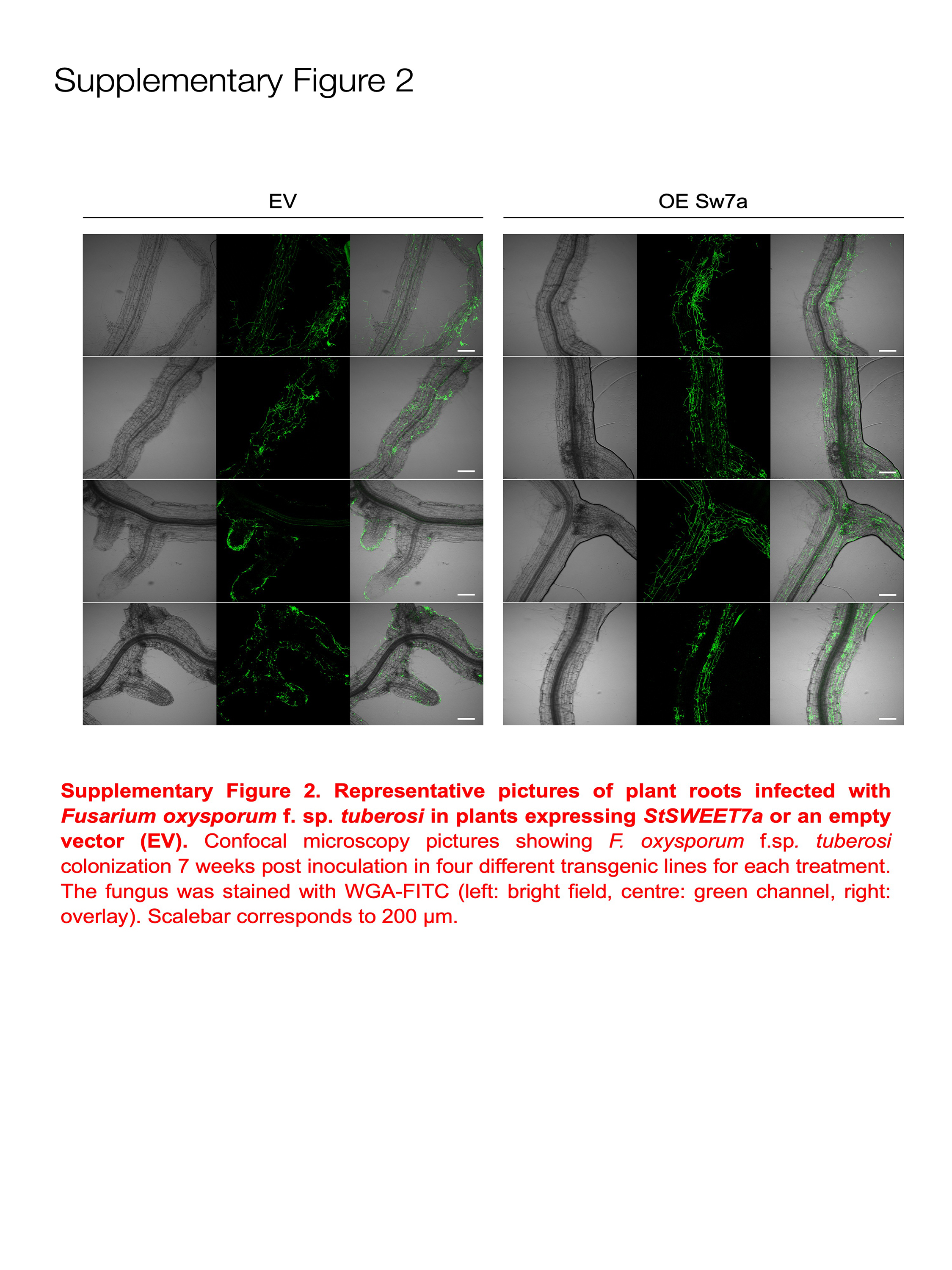

Supplement: Supplementary Figure 2 — Representative pictures of plant roots infected with Fusarium oxysporum f. sp. tuberosi in plants expressing StSWEET7a or an empty vector (EV). Confocal microscopy pictures showing F. oxysporum f. sp. tuberosi colonization 7 weeks post inoculation in four different transgenic lines for each treatment. The fungus was stained with WGA-FITC (left, bright field; center, green channel; right, overlay). Scale bar corresponds to 200 μm. [file Image_2.jpeg]
