# Supplementary material for: Overexpression of the Potato Monosaccharide Transporter StSWEET7a Promotes Root Colonization by Symbiotic and Pathogenic Fungi by Increasing Root Sink Strength
Source: Front Plant Sci. 2022 Mar 24;13:837231. doi: 10.3389/fpls.2022.837231 (PMC8987980; doi:10.3389/fpls.2022.837231)
Supplement: Supplementary file 3 [file Table_1.docx]

**Supplementary Table S1**

Primers used in this study

| **Cloning of promoter-reporter constructs via Gateway cloning** | | |
| --- | --- | --- |
| Gene name | Primer forward | Primer reverse |
| *StSWEET1b* | CACCGAATAAAATAGTCACTGGGGCAC | TCATACCAAAAATCCCAAACAAAG |
| *StSWEET2c* | CACCCCTGGTATTACTTCATG | CAAATTTTGACACCACAAAAATCAATC |
| *StSWEET7a* | CACCCAATTAACTCTAGTAAAATAG | GTCAATGAAGAAAAAGAGAGGAAC |
| *StSWEET12a* | CACCGTATCAGAATATACTCCTCCG | ATTCTCTTCTAAACAGAACAAATAAAC |
|  |  |  |
| **Yeast expression analysis** | | |
| Gene name | Primer forward | Primer reverse |
| *StSWEET1b* | ctgcagATGGCAATCATCAAAATTCTCCACAC | ctcgagTCATACTTTTGCATCACCACTTTGG |
| *StSWEET2b* | atctgcagCAAACATATCTTTTAAGCAAAAATGGA | ttctcgagTTCATGCATTGGACTCCAAGA |
| *StSWEET2c* | tactcgagGATGGTTTCTTTGGCTTCGTC | aactcgagGATCTCTAGTTCATGAATAAGACAC |
| *StSWEET7a* | tactgcagATGGTTTTAGATAGAGAGCATGC | attctcgagTTAAACACTAGCCCCGTTGC |
| *StSWEET12a* | ctgcagATGATGACTCATTTGGCTTTTGTA | ctcgagTTAGGAGACCTCTATGATTGC |
|  |  |  |
| **Localization analysis in *Nicotiana benthamiana*** | | |
| Gene name | Primer forward | Primer reverse |
| *StSWEET1b* | CACCATGGCAATCATCAAAATTCTCCAC | TACTTTTGCATCACCACTTTGG |
| *StSWEET2b* | CACCATGGAAATTTCTGGAGCAG | TGCATTGGACTCCAAGAGAGG |
| *StSWEET2c* | CACCATGGTTTCTTTGGCTTCGTC | TGAATAAGACACTATAAGGCCTTCG |
| *StSWEET7a* | CACCATGGTTTTAGATAGAGAGCATGC | AACACTAGCCCCGTTGCTC |
| *StSWEET12a* | CACCATGATGACTCATTTGGCTTTTGTA | GGAGACCTCTATGATTGCCTTTG |
|  |  |  |
| **Overexpression analysis in *Solanum tuberosum*** | | |
| Gene name | Primer forward | Primer reverse |
| *StSWEET7a* | CACCATGGTTATTGATAGAGAGCATGC | TTAGAATATGGGCCTTCCAAAC |
|  |  |  |
|  |  |  |
|  |  |  |
|  |  |  |
| **Quantitative Real Time PCR** | | |
| Gene name | Primer forward | Primer reverse |
| *FoTEF* | CGGTAAGGGTTCCTTCAAGT | TGACCGGGAGCGTCGATGA |
| *RiMST2* | GGCAGGATATTTGTCTGATAG | GCAATAACTCTTCCCGTATAC |
| *RiOLE1-like* | TATGTGTTGGATGTTTCGTCA | CATAGCCGTCATGGTATTGG |
| *RiTEF* | TGTTGCTTTCGTCCCAATATC | GGTTTATCGGTAGGTCGAG |
| *StActin* | AGCAGCATGAAGATTAAGGTTGT | AATCCACATCTGCTGGAAGGT |
| *StFatM* | GTCAAAGGCCATTTAGCTATACAC | CTCTTCCAAGTAGTCCTTCCTCTAAC |
| *StInvCD141* | GATACTGTTGATAACGATGTGAG | GATTTCAACCTTTTCTCCCTTGT |
| *StPT4* | ATGAAGGGAAGCCATTTGATG | TACCTTTCCCATGTTAATCGC |
| *StSWEET1a* | GTGTTCTTATGTGGCGCTTC | TCAACGACTCATCAAAGGTTG |
| *StSWEET1b* | TTGTTTTTCTTTGTGGCACCTC | ACTCTTCCATCTTCCTCACC |
| *StSWEET1c* | TACCAAATTCAGTCGGATCTC | CCTTCACCCTTTCTTATACAC |
| *StSWEET1d* | TTCATCTTCGCCCTGCTC | CGCGTCTTCGTTGTAAGC |
| *StSWEET1e* | CTGTCGTTTGCTGTCGTG | ACTCATCCACAGCCGTTG |
| *StSWEET1f* | CATTTGCCGCTATCTTATCTTG | TCTGCTGCGACTCTTCAAC |
| *StSWEET1g* | CATTTGCTGTTGTCGTATCTTG | CAACTGTACTATTCCTAAAGCC |
| *StSWEET2a* | CATTTTATCTATCCCTTGCGAC | AGAGTCCAAGAGAGGTGC |
| *StSWEET2b* | ATGTTTGCTTCTCCGTTGTTC | AGGTCTTGTTGGCTCCTC |
| *StSWEET2c* | ATCAGGACCAGGAGTGTTG | TCCTCCCTTGAAGAATTTCTG |
| *StSWEET3* | GGACTACTCAGCCATGATC | CTCAGTGACAACGAGCATC |
| *StSWEET5a* | ATTCTGGCAACCGCCTTC | GAACTAAAGCTAATAGTGCTCC |
| *StSWEET5b* | GATCAGCACCAAGAGTGTG | TTCATCGTCCCAGTTGGTG |
| *StSWEET7a* | AGTACATGCCCTTCTATCTTTC | TGTCTTTCTGCTATCTGTCTCC |
| *StSWEET7b* | CATGAAACTGGTGATCAAGAC | CTCCTTTAGCCTCTCTTGC |
| *StSWEET7c* | CACATTTATCAATTGTGGGATTTG | CTTTCGTCCTTTGTTTACGATC |
| *StSWEET7d* | GCTTCTCCTTTGGCTGTC | GTGCAGTTCCCATAGAATTTG |
| *StSWEET10a* | CAGCGGTAATTGTGTCGG | TTGGGTATGTTTTCTACATTAGG |
| *StSWEET10b* | CTTGCTCAAAGATATTAACATTGC | CAACCTGTGTTTTATCTGAGC |
| *StSWEET10c* | CAAAAGGCTGAAGTCATTGTG | GCAACTTGGGCACATTCTC |
| *StSWEET10d* | GCGGTGATGTGGTTCTTC | TTGCAGATTGGTTAGCTTCAC |
| *StSWEET10e* | CAACAAGTTCTACCGGACC | GTTAGTGCAACTACATCTTGC |
| *StSWEET11a* | GGAATACTTCAAATGGTGCTC | CAGACACGACGCAATGTG |
| *StSWEET11b* | AACCTGAAGTCATTGTGAAGG | CAGTAACAACCAGTAAACCTG |
| *StSWEET11c* | TGGATTTGCCTTGTGTTTTCC | ACCCAACACATTTGGAATAGC |
| *StSWEET11d* | ACGTTTGTAGCACCATTAGG | CACCATTTGGACCACACC |
| *StSWEET12a* | TGGTCTACTACGCAAAGATTAC | CTCTTCATCAACAACAGTTTCTG |
| *StSWEET12b* | TTCTAAGTCAAACAACGTCGC | GGGAATCTCCAAGAATTTTACC |
| *StSWEET12c* | CAAGAGCAGCCCACAAAAG | GGAATCTTCAAGATTTTTAGCATC |
| *StSWEET12d* | GGATCAAGAGCTGCCCC | TGTCTTCCCAAGCTTTTGAAG |
| *StSWEET12e* | GTATGGAATTTACCGAAACGC | CACTTGTGCTAGTTGCTCG |
| *StSWEET12f* | AGAGTTTGTAAAGGAGCAGAATC | TATTTCCAGTGTTTTCCGCAG |
| *StSWEET17a* | CTC CCC TTT TGG TGT TGT G | AAG CAT CCC GTC CCA TTT G |
| *StSWEET17b* | GTGAGAAGTAAGAGTGTTGAG | TCCAAGAAACCATCCAATTCC |
| *StSWEET17c* | GCTTATGCAGTTCTTGTCAAAG | TTTCATGGGCTCCATTAACATC |
| *StTEF1* | GAACATCCATTGCTTGCTTTC | CACAATTTCATCGTACCTAGC |
