# Supplementary material for: Overexpression of the Potato Monosaccharide Transporter StSWEET7a Promotes Root Colonization by Symbiotic and Pathogenic Fungi by Increasing Root Sink Strength
Source: Front Plant Sci. 2022 Mar 24;13:837231. doi: 10.3389/fpls.2022.837231 (PMC8987980; doi:10.3389/fpls.2022.837231)
Supplement: Supplementary file 4 [file Table_2.docx]

Supplementary Table S2

| EXPERIMENT 1 | | |  | EXPERIMENT 2 | | |
| --- | --- | --- | --- | --- | --- | --- |
|  |  |  |  |  |  |  |
| Root fresh weight | | |  | Root fresh weight | | |
| Myc- | EV | ab |  | Myc- | EV | a |
|  | *Sw7a* OE | a |  |  | *Sw7a* OE | a |
| Myc+ | EV | b |  | Myc+ | EV | a |
|  | *Sw7a* OE | a |  |  | *Sw7a* OE | a |
|  |  |  |  |  |  |  |
| Shoot fresh weight | | |  | Shoot fresh weight | | |
| Myc- | EV | a |  | Myc- | EV | a |
|  | *Sw7a* OE | a |  |  | *Sw7a* OE | ab |
| Myc+ | EV | a |  | Myc+ | EV | b |
|  | *Sw7a* OE | a |  |  | *Sw7a* OE | b |
|  |  |  |  |  |  |  |
| Shoot Pi | | |  | Shoot Pi | | |
| Myc- | EV | a |  | Myc- | EV | a |
|  | *Sw7a* OE | a |  |  | *Sw7a* OE | a |
| Myc+ | EV | a |  | Myc+ | EV | a |
|  | *Sw7a* OE | a |  |  | *Sw7a* OE | a |
|  |  |  |  |  |  |  |
| Root Sw7a rel. expr. | | |  | Root Sw7a rel. expr. | | |
| Myc- | EV | a |  | Myc- | EV | a |
|  | *Sw7a* OE | ab |  |  | *Sw7a* OE | b |
| Myc+ | EV | a |  | Myc+ | EV | c |
|  | *Sw7a* OE | b |  |  | *Sw7a* OE | b |
|  |  |  |  |  |  |  |
| Root *StInvCD141* rel. expr. | | |  | Root *StInvCD141* rel. expr. | | |
| Myc- | EV | a |  | Myc- | EV | a |
|  | *Sw7a* OE | ab |  |  | *Sw7a* OE | a |
| Myc+ | EV | ab |  | Myc+ | EV | b |
|  | *Sw7a* OE | b |  |  | *Sw7a* OE | ab |

**Supplementary Table S2.** Statistical analysis per experiment of the four treatments (EV non-mycorrhizal, *StSWEET7a* OE non-mycorrhizal, EV mycorrhizal, *StSWEET7a* OE mycorrhizal) together. Statistical significance was calculated, depending on the normality of the samples, either by using the one-way analysis of variance (ANOVA) or the Kruskal-Wallis test plus the corresponding post hoc test as specified in Materials and Methods. Different letters indicate significant differences for a *p*-value > 0.05.
