# Supplementary material for: Overexpression of the Potato Monosaccharide Transporter StSWEET7a Promotes Root Colonization by Symbiotic and Pathogenic Fungi by Increasing Root Sink Strength
Source: Front Plant Sci. 2022 Mar 24;13:837231. doi: 10.3389/fpls.2022.837231 (PMC8987980; doi:10.3389/fpls.2022.837231)
Supplement: Supplementary file 5 [file Table_3.docx]

Supplementary Table S3

| NON-MYCORRHIZAL | | |  | MYCORRHIZAL | | |
| --- | --- | --- | --- | --- | --- | --- |
|  |  |  |  |  |  |  |
| Root fresh weight | | |  | Root fresh weight | | |
| EXP1 | EV | a |  | EXP1 | EV | a |
|  | *Sw7a* OE | a |  |  | *Sw7a* OE | b |
| EXP2 | EV | a |  | EXP2 | EV | b |
|  | *Sw7a* OE | a |  |  | *Sw7a* OE | b |
|  |  |  |  |  |  |  |
| Shoot fresh weight | | |  | Shoot fresh weight | | |
| EXP1 | EV | a |  | EXP1 | EV | a |
|  | *Sw7a* OE | a |  |  | *Sw7a* OE | ab |
| EXP2 | EV | b |  | EXP2 | EV | b |
|  | *Sw7a* OE | b |  |  | *Sw7a* OE | b |
|  |  |  |  |  |  |  |
| Shoot Pi | | |  | Shoot Pi | | |
| EXP1 | EV | a |  | EXP1 | EV | a |
|  | *Sw7a* OE | a |  |  | *Sw7a* OE | a |
| EXP2 | EV | a |  | EXP2 | EV | ab |
|  | *Sw7a* OE | a |  |  | *Sw7a* OE | b |
|  |  |  |  |  |  |  |
| Root *StSWEET7a* rel. expr. | | |  | Root *StSWEET7a* rel. expr. | | |
| EXP1 | EV | a |  | EXP1 | EV | a |
|  | *Sw7a* OE | a |  |  | *Sw7a* OE | ab |
| EXP2 | EV | a |  | EXP2 | EV | a |
|  | *Sw7a* OE | b |  |  | *Sw7a* OE | b |
|  |  |  |  |  |  |  |
| Root *StInvCD141* rel. expr. | | |  | Root *StInvCD141* rel. expr. | | |
| EXP1 | EV | a |  | EXP1 | EV | a |
|  | *Sw7a* OE | ab |  |  | *Sw7a* OE | ab |
| EXP2 | EV | b |  | EXP2 | EV | c |
|  | *Sw7a* OE | b |  |  | *Sw7a* OE | bc |

**Supplementary Table S3.** Statistical analysis per mycorrhizal treatment of the four treatments (EV Exp1, *StSWEET7a* OE Exp1, EV Exp2, *StSWEET7a* OE Exp2) together. Statistical significance was calculated, depending on the normality of the samples, either by using the one-way analysis of variance (ANOVA) or the Kruskal-Wallis test plus the corresponding post hoc test as specified in Materials and Methods. Different letters indicate significant differences for a *p*-value > 0.05.
